# Permanent Draft Genome Sequence of *Desulfurococcus amylolyticus* Strain Z-533^T^, a Peptide and Starch Degrader Isolated from Thermal Springs in the Kamchatka Peninsula and Kunashir Island, Russia

**DOI:** 10.1128/genomeA.00078-17

**Published:** 2017-04-13

**Authors:** Dwi Susanti, Eric F. Johnson, Alla Lapidus, James Han, T. B. K. Reddy, Supratim Mukherjee, Manoj Pillay, Anna A. Perevalova, Natalia N. Ivanova, Tanja Woyke, Nikos C. Kyrpides, Biswarup Mukhopadhyay

**Affiliations:** a Department of Biochemistry, Virginia Tech, Blacksburg, Virginia, USA; b Centre for Algorithmic Biotechnology, ITBM, St. Petersburg State University, St. Petersburg, Russia; c U.S. Department of Energy Joint Genome Institute, Walnut Creek, California, USA; d Biological Data Management and Technology Center, Lawrence Berkeley National Laboratory, Berkeley, California, USA; e Virginia Bioinformatics Institute, Virginia Tech, Blacksburg, Virginia, USA; f Winogradsky Institute of Microbiology, Research Center of Biotechnology Russian Academy of Sciences, Moscow, Russia; g Department of Biology, Faculty of Science, King Abdulaziz University, Jeddah, Saudi Arabia; h Department of Biological Sciences, Virginia Tech, Blacksburg, Virginia, USA

## Abstract

*Desulfurococcus amylolyticus* Z-533^T^, a hyperthermophilic crenarcheon, ferments peptide and starch, generating acetate, isobutyrate, isovalerate, CO_2_, and hydrogen. Unlike *D. amylolyticus* Z-1312, it cannot use cellulose and is inhibited by hydrogen. The reported draft genome sequence of* D. amylolyticus* Z-533^T^ will help to understand the molecular basis for these differences.

## GENOME ANNOUNCEMENT

*Desulfurococcus  amylolyticus* Z-533^T^ (DSM 3822^T^), an inhabitant of thermal springs in the Kamchatka Peninsula and Kunashir Island, Russia, is a hyperthermophilic, anaerobic, sulfur-reducing crenarcheon (1). It is a nonmotile regular coccus of 0.7 to 1.5 μm in diameter (1). In laboratory cultures, *D. amylolyticus* Z-533^T^ uses peptides such as casein hydrolysates, peptone, and yeast extract and simple carbohydrates that include starch, pectin, and glycogen as energy substrates. Sulfur stimulates growth, and, when it is employed, H_2_S is produced. The absence of sulfur results in poor growth and H_2_ production.

The 16S rRNA gene sequences of *D. amylolyticus* Z-533^T^ differ by 0.1 to 0.3% from those of other *D. amylolyticus* strains, namely, Z-1312 and 1221n (2–4), which were formerly known as *D. fermentans* Z-1312 and *D. kamchatkensis* 1221n, respectively (5). Of these, only *D. amylolyticus* Z-1312 degrades cellulose (2) and is not inhibited by the presence of hydrogen (6). However, it lacks known cellulose genes and might employ novel mechanisms for cellulose degradation. Thus, a genomic analysis of three desulfurococci will give insight into the mechanisms by which *D. amylolyticus* Z-1312 degrades cellulose and by which other strains are inhibited by hydrogen.

The permanent draft genome of* D. amylolyticus* strain Z-533^T^ (DSM 3282^T^) was generated at the DOE Joint Genome Institute (JGI) (7). The term “permanent” indicates that this genome sequence has been completed at a draft level and submitted to GenBank (8). The sequencing of a standard shotgun library on the Illumina platform generated 28,000,000 reads of 150 bp. These raw sequences were passed through DUK (9), and filtered reads were assembled with Velvet (10) and ALLPATHS-LG (11, 12). The final assembly contained one scaffold and two contigs. Structural and functional annotations were performed using the JGI’s microbial genome annotation pipeline (13). The predicted coding sequences were translated and used to search the National Center for Biotechnology Information (NCBI) nonredundant, UniProt, TIGR-Fam, Pfam, PRIAM, KEGG, COG, and InterPro databases. Ribosomal and tRNA genes were identified with HMMER version 3.0rc1 (14) and tRNAscan-SE version 1.23 (15), respectively. Noncoding genes were predicted using Infernal version 1.0.2 (16). Additional annotation was performed within the Integrated Microbial Genomes—Expert Review platform (17). Clustered regularly interspaced short palindromic repeats (CRISPR) elements were detected using CRT (18) and PILER-CR (19).

The draft assembly of *D. amylolyticus* Z-533^T^ resulted in one scaffold and two contigs with a total sequence size of 1,309,099 bp and a 45.2% G+C content. It encoded 1,394 polypeptides, 59 RNAs (one 5S-, one 16S-, one 23S rRNA, 47 tRNAs, and nine other RNAs), and one putative CRISPR. The analysis predicted a peptide and starch degradation system for *D. amylolyticus* Z-533^T^ that is present in *D. amylolyticus* 1221n and Z-1312.

### Accession number(s).

This whole-genome shotgun project has been deposited at DDBJ/ENA/GenBank under the accession number AZUU00000000. The version described in this paper is the first version, AZUU01000000.
